# Identification and Characterization of a Novel Microalgal Strain from the Antofagasta Coast *Tetraselmis marina* AC16-MESO (Chlorophyta) for Biotechnological Applications

**DOI:** 10.3390/plants12193372

**Published:** 2023-09-25

**Authors:** Maria Teresa Mata, Henry Cameron, Vladimir Avalos, Carlos Riquelme

**Affiliations:** 1 Department of Biotechnology, Faculty of Marine Sciences and Biological Resources, University of Antofagasta, Antofagasta 1240000, Chile; 2 Centro de Bioinnovación de Antofagasta (CBIA), Faculty of Marine Sciences and Biological Resources, University of Antofagasta, Antofagasta 1240000, Chile; henry.cameron@uantof.cl (H.C.); vladimir.avalos@uantof.cl (V.A.); carlos.riquelme@uantof.cl (C.R.)

**Keywords:** biodiversity, chlorophyta, identification, biotechnology

## Abstract

The wide rocky coastline of the Antofagasta hosts an intertidal ecosystem in which the species that inhabit it are routinely exposed to a wide range of physical and chemical conditions and have therefore evolved to tolerate extremes. In the search for new species of potential biotechnological interest with adaptations to a wide range of environmental conditions, the isolation and characterization of microalgae from these ecosystems is of great interest. Here, a new microalgal strain, *Tetraselmis marina* AC16-MESO, is described, which was isolated from a biofilm collected on the intertidal rocks of the Antofagasta coast (23°36′57.2″ S, 70°23′33.8″ W). In addition to the morphological characterization, 18S and ITS sequence as well as ITS-2 secondary structure analysis revealed an identity of 99.76% and 100% with the species *Tetraselmis marina*, respectively. The analyses of the culture characteristics and biochemical content showed similarities with other strains that are frequently used in aquaculture, such as the species *Tetraselmis suecica*. In addition, it is tolerant of a wide range of salinities, thus allowing its culture in water of varying quality. On the other hand, added to these characteristics, the results of the improvement of the lipid content in stressful situations of salinity observed in this study, together with other antecedents such as the potential in bioremediation already published for this strain by the same research group, present a clear example of its biotechnological plasticity. It is noteworthy that this strain, due to its characteristics, allows easy collection of its biomass by decantation and, therefore, a more cost-efficient harvesting than for other microalgal strains. Therefore, this new strain of *Tetraselmis marina*, first report of this species in Chile, and its morphologically, molecularly and biochemically description, presents promising characteristics for its use in biotechnology and as feed for aquaculture.

## 1. Introduction

The current interest in microalgae goes well beyond their simple use as a foodstuff for animals and humans, which was first proposed more than five decades ago [1]. Advances in processing technologies have enabled the use of algal biomass in the production of fine chemicals and pharmaceuticals, including amino acids, vitamins, carotenoids, fatty acids, polysaccharides, and antibiotics [2,3,4]. The past few years have also seen the development of various processes making use of microalgal biomass as a source of renewable energies, such as biofuel and biogas [5,6,7,8,9]. Microalgae are also widely used as fertilizers [2,10,11] and in environmental mitigation, including secondary sewage treatment [2,12,13] and bioremediation [14,15,16,17]. From an economic perspective, several species of the genus *Tetraselmis* (Chlorodendrophyceae, Chlorophyta) have an important commercial potential due to their euryhaline and eurythermal nature; *Tetraselmis* spp. can thus be cultured outdoors within a wide range of salinities [18,19]. The use of seawater or other saltwater greatly improves the economic and environmental sustainability of this potential resource [20,21,22]. It furthermore reduces the risk of contamination with other species and might thus make the culture more reliable in the long term [19]. In aquaculture, the genus *Tetraselmis* has traditionally been used as feed for juvenile mollusks, such as clams, oysters, and abalones, and for the larvae of other marine organisms, such as prawns, shrimps, and rotifers [23,24,25]. Strains high in lipids additionally harbor the potential for the production of biocombustibles, such as biodiesel [18,19,26]. An important fraction of *Tetraselmis* sp. biomass consists of carbohydrates; this fraction increases with rising salinity of the culture medium [27]. Likewise, *Tetraselmis* sp. is known to produce large quantities of carotenoids and vitamin E [28]. There are also studies of its usefulness for bioremediation of aquaculture wastewater [29,30,31]. In addition to these advantages, *Tetraselmis* sp. settles spontaneously and can thus be harvested by decantation with relative ease and at high profit [32,33].

The genus *Tetraselmis* comprises 33 accepted species names; 1 accepted variety, and 1 accepted forma in the database at present [34], including several taxa previously assigned to the genera *Platymonas*, *Prasinocladus*, and *Aulacochlamys* [18]. These species are unicellular green algae 10–20 µm in size, ellipsoid to ovoid, generally mobile with four identical flagella emerging from an anterior cleft in the cell. Generally, they have a single chloroplast with one eyespot and one pyrenoid. The cells are usually covered by a thecal wall formed by the fusion of extracellular scales, which does not contain silica or calcium carbonate. They do, however, have a wall fundamentally consisting of carbohydrates, mainly 2-keto sugar acid, characteristic of the Prasinophyceae [18,35]. Some species form vegetative cysts with thick cell walls [36,37,38]. While the most well-known species of the genus are mobile unicellular organisms, some species previously classified as *Prasinocladus* spp. are able to form sessile colonies during certain stages of their life cycles [37,38,39]. The cells reproduce asexually by simple division of the mother cell, commonly giving rise to two daughter cells in mirror positions. Sexual reproduction is not known in this genus [25,40]. Carbohydrates are accumulated within the cell in the light and consumed in cell division during the dark. Although cell division predominantly happens in the dark, the separation of the daughter cells is light-induced [35]. Most *Tetraselmis* spp. are planktonic or benthic, and some can be found in freshwater habitats [41]. Some species have been described as endosymbionts of marine animals, e.g., *Tetraselmis convolutae* and a non-described species which has been isolated from the radiolarian *Spongodrymus* [18,42,43,44].

While the morphology and ultrastructure of many *Tetraselmis* spp. have been characterized, there are still taxonomical ambiguities within the genus [25]. Due to the complexities of the classical method of cellular characterization under the microscope, the identification at the species level is arduous. The described species mainly differ from each other in cell size and form, the presence and morphology of the chloroplast, and the position of the eyespot. Most of these characteristics do not serve as good descriptors, thus complicating the taxonomy of the genus [25]. Molecular techniques, such as DNA sequence analysis, therefore, represent a better, quicker, and more reliable complementary tool for species identification and delineation [18,25]. Molecular studies seeking to establish species identity or phylogenetic relationships within the genus *Tetraselmis* are relatively recent and have mostly been based on SSU rDNA sequences [18,25,45]. In Chile, the presence of *Tetraselmis suecica* and *Tetraselmis tetrathele* has been registered on the coast of Valparaiso [46] based on morphological characteristics. In addition, three new *Tetraselmis* strains were reported from Dichato and the Coliumo Peninsula in the Biobío Region, and from Caldera in the Atacama Region [25].

The coast of Antofagasta, Chile, is characterized by a wide rocky shore, which harbors an intertidal ecosystem with the frequent occurrence of biofilms mainly consisting of algal–bacterial consortia. This ecosystem is particularly interesting because of the wide range of physical and chemical conditions to which the organisms inhabiting it are exposed. As a consequence, these organisms have evolved a tolerance and pre-acclimatization to extreme conditions in terms of temperature changes, salinity, pH, nutrient availability, and solar irradiation, amongst others, all of which make them highly interesting species. In the search for new species with adaptations to a wide range of endemic conditions for use in biotechnology, the isolation and characterization of microalgal species from these biofilms is thus of interest. Here, a novel strain of *Tetraselmis* was isolated from a biofilm collected on the intertidal rocks of the Antofagasta coast (23°36′57.2″ S, 70°23′33.8″ W). Molecular sequence markers (SSU rDNA and internal transcribed spacers (ITS)) were utilized, as well as ITS-2 secondary structures to identify the strain. Finally, the morphological characteristics, growth, biochemical composition, and salinity tolerance of microalgae, as well as lipid content under saline stress, were investigated with the aim of exploring potential applications in biotechnology.

## 2. Results

### 2.1. Morphological Characterization of the Strain AC16-MESO

**Figure 1:**
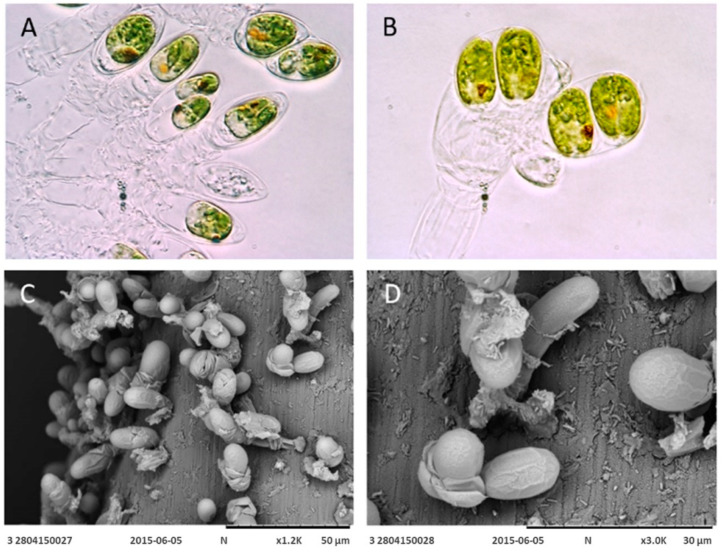
Bright-field image (**A**,**B**) at 100× magnification and scanning electron micrograph (**C**,**D**) of the new microalgal strain *T. marina* AC16-MESO fixed to a mesh type substrate.

Spreading the samples on agar plates resulted in the growth of different colonies, which were isolated according to their color and growth characteristics. Colonies of the strain AC16-MESO were of an intense green and grew well both on solid and in liquid f/2 medium. Bright-field microscopy revealed ellipsoid cells of 15–20 µm length and 5–10 µm width, with a basal pyrenoid and a clearly visible eyespot in varying positions (Figure 1A,B). Cells divided in asexual reproduction, resulting in two daughter cells in mirror positions (Figure 1B). Cells were in a persistent benthic state with colonies forming on a septate pedunculus (Figure 1A,B). Transmission microscopy revealed how these structures allow the microalga to adhere the substrate and showed that the cells are covered by a theca resulting from the fusion of extracellular scales (Figure 1C,D).

### 2.2. Analysis of 18S Ribosomal DNA and the ITS1-5.8-ITS2 Region

Sequencing and assembling resulted in a 1617 bp consensus sequence of the 18S ribosomal locus and a 612 bp consensus sequence for the ITS1-5.8-ITS2 locus. BLASTing the 18S consensus sequence against the NCBI GenBank database found a 99.76% identity with the species *Tetraselmis marina* strain CCMP898 (accession number HE610131.1) and *T. marina* (accession number KY045847.1). The difference was of a four base pair in both cases.

For the ITS1-5.8-ITS2 locus, there was a 100% identity with the species *T. marina* strain IOAC331S (accession number KC800942.1) and 99.84% identity with the species *T. marina* CCMP898 (accession number HE610131.1). The difference with *T. marina* CCMP898 was of a single base pair.

### 2.3. ITS-2 Secondary Structure

**Figure 2:**
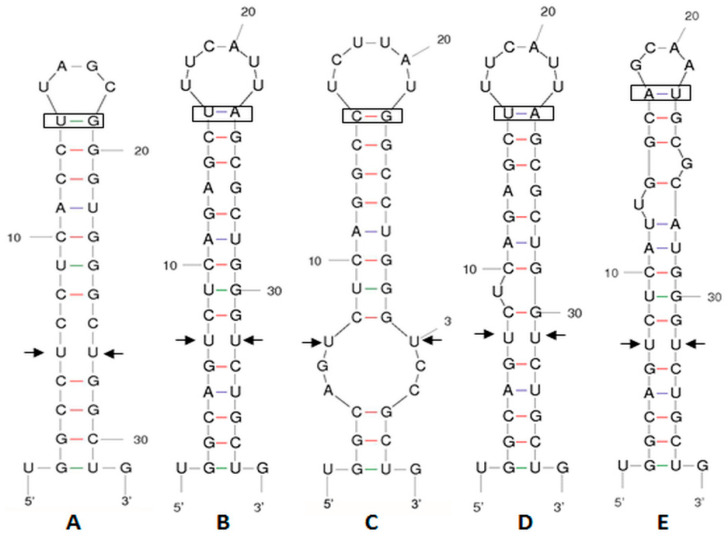
Secondary structure predictions for ITS-2 helix II (5′ to 3′). (**A**) *T. marina* AC16-MESO, *T. marina* IOAC331S, and *T. marina* CCMP898, (**B**) *T. suecica* and *Tetraselmis* sp. CCM-UDEC 109 (from Dichato), (**C**) *T. subcondiformis* and *Tetraselmis* sp. CCM-UDEC 134 (from Caldera), (**D**) *Tetraselmis* sp. CCM-UDEC 114 (from Coliumo), and (**E**) *T. striata*. Each colored interaction brings distinct properties to the intricate folding pattern. The red color emphasizes the robust G-C base pair interactions, crucial for both structural integrity and functional efficacy of the molecule. Blue color markings represent A-U interactions and green markings denote G-U interactions. The arrows indicate the supposed sites of RNA processing, and the boxes represent the compensatory base changes (CBCs).

**Figure 3:**
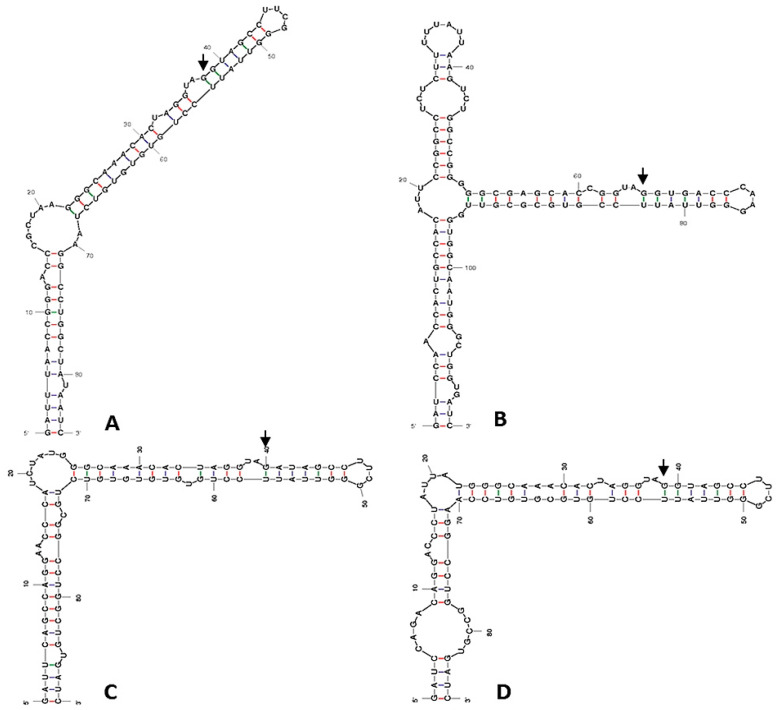
Secondary structure predictions for ITS-2 helix III (5′ to 3′). (**A**) *T. suecica*, *Tetraselmis* sp. CCM-UDEC 109 (from Dichato), and *Tetraselmis* sp. CCM-UDEC 114 (from Coliumo), (**B**) *T. marina* AC16-MESO, *T. marina* IOAC331S and *T. marina* CCMP898. (**C**) *T. subcondiformis* and *Tetraselmis* sp. CCM-UDEC 134 (from Caldera), and (**D**) *T. striata*. The red color emphasizes the robust G-C base pair interactions, crucial for both structural integrity and functional efficacy of the molecule. Blue color markings represent A-U interactions and green markings denote G-U interactions. Arrows indicate potential RNA processing sites.

The results of the analysis of the ITS-2 secondary structure showed that, even in the presence of a 1 bp difference in the DNA sequence between the strain *T. marina* CCMP898 and AC16-MESO, helices II and III of the spacer share the same secondary structure in AC16-MESO and the two *T. marina* strains IOAC331S and CCMP898 (Figure 2A and Figure 3B). *T. suecica* and the Chilean *Tetraelmis.* sp. CCM-UDEC 109 had the same structure for helices II (Figure 2B) and III; the structure of helix III is also shared by the Chilean strain *Tetraelmis* sp. CCM-UDEC 114 (Figure 3A), which does, however, have a different helix II structure (Figure 2D). *T. subcondiformis* and the Chilean *Tetraselmis.* sp. CCM-UDEC 134 share both helix II and III secondary structures (Figure 2C and Figure 3C), and finally, for strain *T. striata*, the secondary structures of both helices II and III are different to those found in all other strains (Figure 2E and Figure 3D).

### 2.4. Phylogenetic Analysis

**Figure 4:**
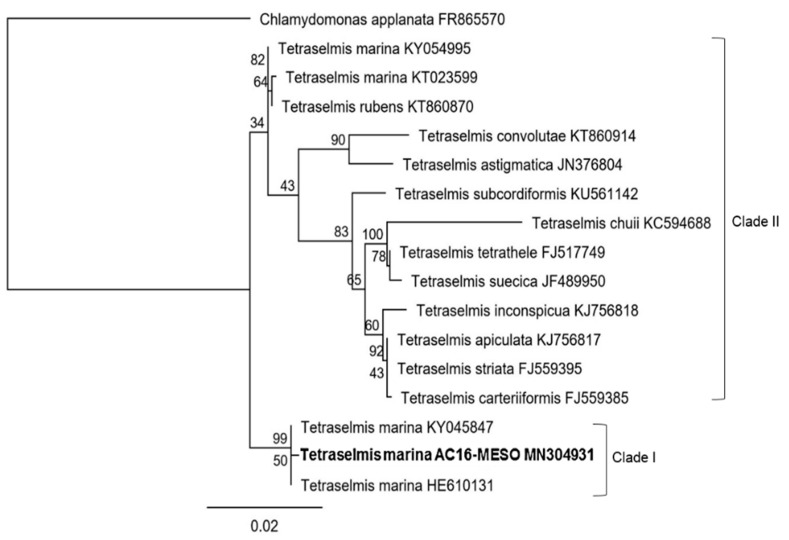
Phylogenetic tree based on 18S sequences of *T. marina* AC16-MESO and related microalgal species.

**Figure 5:**
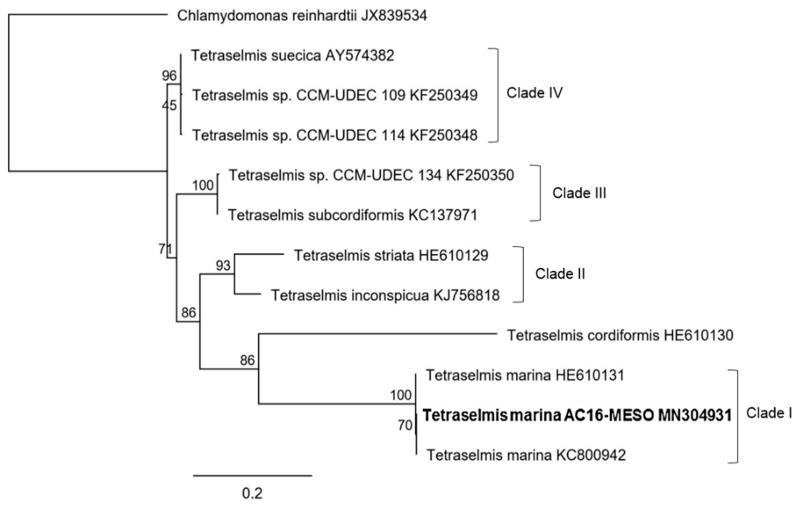
Phylogenetic tree based on ITS1-5.8S-ITS2 sequences of *T. marina* AC16-MESO and related microalgal species.

Both phylogenetic trees of AC16-MESO and strains with highly similar sequences identified in the NCBI GenBank BLAST analysis classify AC16-MESO as part of the family Chlorodendraceae within the genus *Tetraselmis* (Figure 4 and Figure 5); likewise, both trees indicate a close relationship with the species *Tetraselmis marina*. Figure 4 shows the tree based on 18S ribosomal sequences; in it, *T. marina* AC16-MESO, *T. marina* CCMP898 (HE610131), and *T. marina* (KY045847) are grouped together with a bootstrap value of 99; they form clade I (Figure 4). Its sister clade, clade II (bootstrap value 34), comprises the others strain of *T. marina* (KY054995 and KT023599) (bootstrap value 82), and the other species of genus *Tetraselmis*: *T convolutae* and *T. astigmatica* (bootstrap value 90); *T subcordiformis*, *T. chuii*, *T tetrathele*, *T. suecica*, *T. inconspicua*, *T. apiculata*, *T. striata*, and *T. carteriiformis* (bootstrap value 83).

Figure 5 shows the tree based on the sequences of the ITS1-5.8-ITS2 region; here, *T. marina* AC16-MESO forms a clade (bootstrap value 100) together with two strains of *T. marina* CCMP898 (HE610131) and *T. marina* (KC800954); this is part of clade I in which the species *T. cordiformis* is also grouped (bootstrap value 86). Its sister clade, clade II, comprises the species *T. striata* and *T. inconspicua* (bootstrap value 93) and clade III comprises the species *Tetraselmis* sp. CCM-UDE 134 y *T. subcordiformis* (bootstrap value 100). Together, clades I–III form one of the two main branches of the tree. On the other main branch of the tree, the clades IV (bootstrap value 96) include: *T. suecica* and species *Tetraselmis.* sp. CCM-UDEC 109, and *Tetraselmis* sp. CCM-UDEC 114, used in the secondary structure folding analysis of the ITS2 region.

### 2.5. Growth Characteristics in f/2 Medium

**Figure 6:**
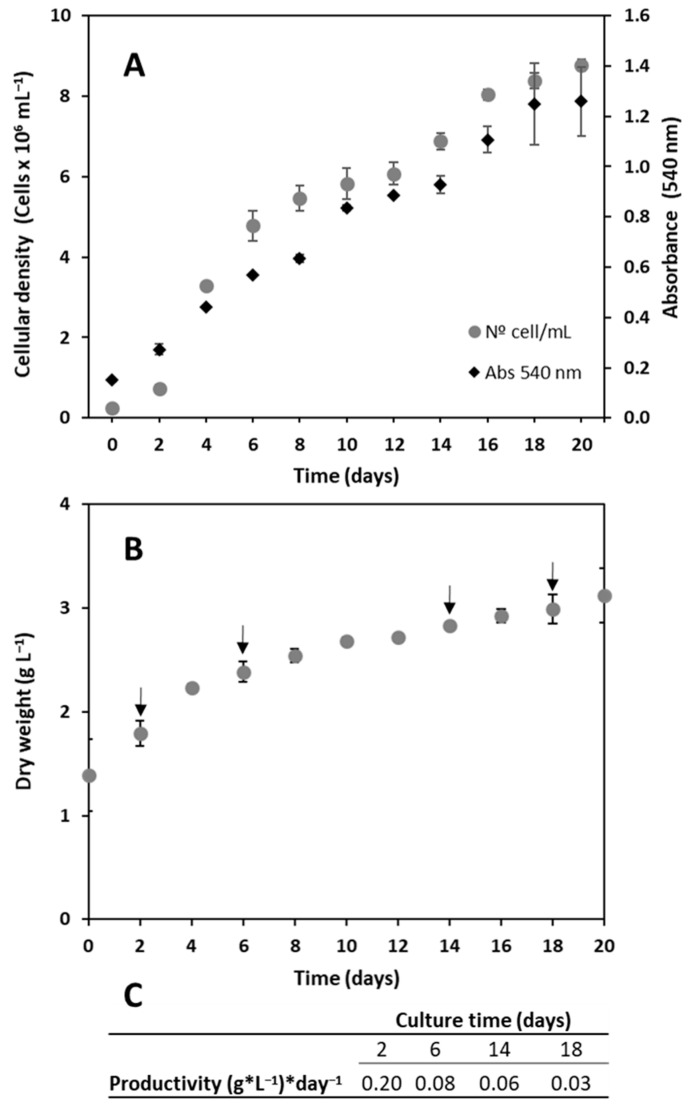
Growth curve of strain *T. marina* AC16-MESO over 20 days expressed as (**A**) cell density (cells × 10^6^/mL) and absorbance at 540 nm, and (**B**) biomass dry weight (g/L). Values represent the mean ± SD of three independent measurements in 2-liter reactors (*n* = 3). (**C**) The table shows the production (g L^−1^ day^−1^) during the exponential phase (day 2), early stationary phase (day 6), stationary phase (day 14), and late stationary phase (day 18). The arrows indicate the times for which biomass production was estimated.

Cellular density (Figure 6) as measured by a direct cell count and by OD540 were positively correlated and followed the equation y = 0.1276x + 0.0791 (R^2^ = 0.95), where x represents cellular density in units of millions of cells and y represents OD540. The cellular density reached a maximum of 8.7 × 10^6^ cells/ml in the stationary phase; the OD540 reached a maximum of 1.3. The maximum productivity was 0.2 g L^−1^ day^−1^, which was reached in the middle of the exponential phase. Upon entering the early stationary phase on day 6 of the culture, productivity decreased to 0.08 g L^−1^ day^−1^, then 0.06 g L^−1^ day^−1^ on day 14, and 0.03 g L^−1^ day^−1^ in the late stationary phase on day 18 of the culture.

### 2.6. Biochemical Characteristic

**Figure 7:**
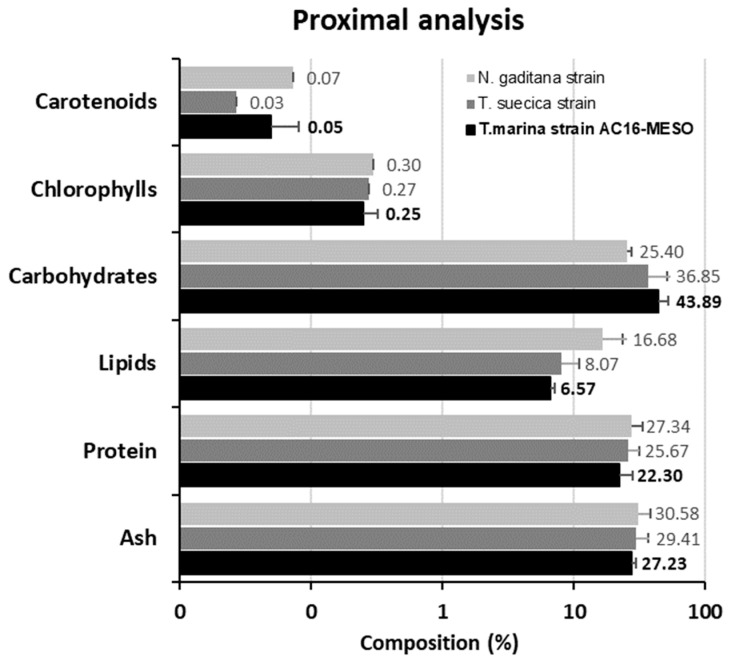
Proximal analysis of *T. marina* AC16-MESO compared with reference biochemical analyses of *N. gaditana* and *T. suecica*. The reference strains were cultivated in the same facilities as the study strain and are part of the collection stock of the Applied Microbiology Unit, University of Antofagasta. Values represent the mean ± SD of three independent measurements (*n* = 3).

The proximate analysis results (Figure 7) demonstrate that the composition of *T. marina* AC16-MESO is similar to that of the other *Tetraselmis* species, particularly *T. suecica*, analyzed here, as well as to that of the strain of *Nannochloropsis gaditana*, used as a reference, except for a higher lipid content in *N. gaditana* (16.68%) compared to *T. marina* AC16-MESO and *T. suecica* (6.57% and 8.07%, respectively). It should be highlighted that the strain AC16-MESO exhibited a higher carotenoid content than *T. suecica* (0.07% vs. 0.03%), similar to *N. gaditana* (0.07%), as well as a higher percentage of carbohydrates for the genus *Tetraselmis* (43.89% for AC16-MESO and 36.85% for *T. suecica*) compared to *N. gaditana* (25.40%). Additionally, *T. marina* AC16-MESO showed a comparable protein content (22.30%) to that of *T. suecica* (25.67%) and *N. gaditana* (27.34%).

The fatty acid composition analysis of the novel strain showed that it is rich in C16, 16:1n7, 16:2n4, 16:3n4, 18:1n9, 18:1n7, 18:2n6, 18:3n3, 18:4n3, 20:1n9, 20:4n6, and 20:5n3. The most abundant fatty acids were palmitic acid (C16; 22.5% of the total fatty acid content), linoleic acid (18:2n6; 11.2%), linolenic acid (18:3n3; 9.1%), oleico acid (18:1n9; 8.8%), stearidonic acid (18:4n3; 7.9%), and EPA (20:5n3; 6.1%). The fatty acid composition is shown in Table 1.

### 2.7. Salinity Tolerance

**Figure 8:**
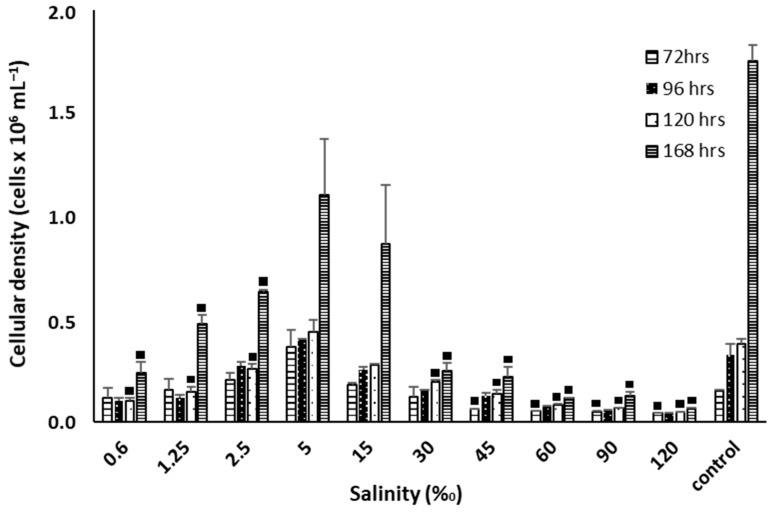
Cellular growth of *T. marina* AC16-MESO at different salt concentrations of 0.6, 1.25, 2.5, 5, 15, 30, 45, 60, 90, 120, and 35 (control) (‰). Squares (■) indicate significant negative differences in the growth respect to the control, with a confidence level of 95%, *n* = 3, *p* < 0.05.

*T. marina*. AC16-MESO was exposed to different salinities ranging from 0.6 to 120‰. The highest cellular densities were found for 2.5‰ (6.3 × 10^5^ cells/mL), 5‰ (1.1 × 10^6^ cells/mL), and 15‰ (8.6 × 10^5^ cells/mL) and was similar to the control (35‰). At lower (0.6–1.25‰) and higher salt concentrations (30–120‰), the cellular density was significantly affected. At 168 hours, the cell density values were lower than the control for all salinity conditions, and the differences in cell density were statistically significant (*p* < 0.05) (Figure 8).

In the same way, the optimal quantum yield (Fv/Fm) of the novel strain *T. marina* AC16-MESO was similar to the control at 2.5‰, 5‰, and 15‰. At lower (0.6–1.25‰) and higher salt concentrations (30–120‰), the Fv/Fm was lower than for the control over 168 h (Table 2).

### 2.8. Neutral Lipid Content Measured by Nile Red

**Figure 9:**
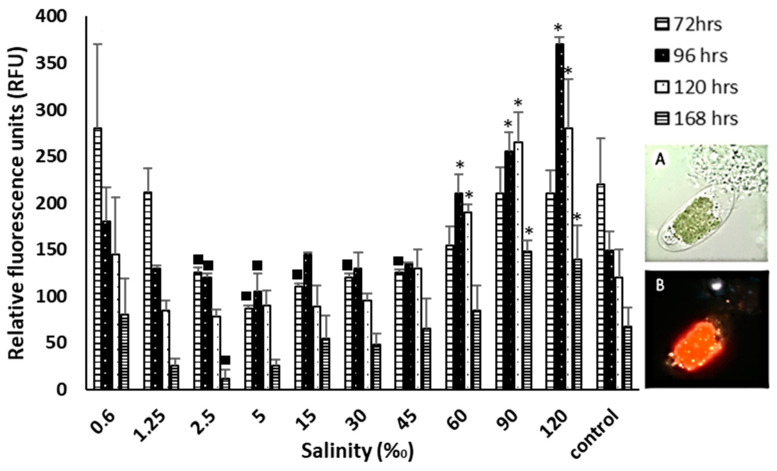
Neutral lipid fluorescence of the strain *T. marina* AC16-MESO with Nile Red expressed in relative fluorescence units (RFU) and micrograph detail (100× magnification) of the microalgae stained. (**A**) Bright-field image and (**B**) UV micrograph of the microalgae stained with Nile Red at 35‰ salinity (control). Asterisks (*) indicate significant positive differences in the RFU with respect to the control; squares (■) indicate significant negative differences in the RFU with respect to the control, with a confidence level of 95%, *n* = 3, *p* < 0.05.

Relative fluorescence units (RFUs) are positively correlated with lipid accumulation. In Figure 9, the location of lipids stained with Nile red in the cell interior is observed. At 0.6‰ salinity, RFU values were higher for *T. marina* AC16-MESO than the control, with a 32% increase at 72 h, 23% at 96 h, 21% at 120 h, and 26% at 168 h. Likewise, for 60‰, 90‰, and 120‰ salinity, the increases over the control were of 33%, 71%, and 138%, respectively, at 96 h; 60%, 124%, and 137% at 120 h, and 37%, 108%, and 97% at 168 h. On the other hand, at 1.25‰, no different differences were observed and between 2.5 and 45‰ salinity, the RFUs were lower than for the control (Figure 9).

## 3. Discussion

Located at the interface of ocean, atmosphere, and terrestrial environments, the intertidal ecosystem is characterized by frequent fluctuations in temperature, ion concentrations, humidity, UV radiation, and the activity of waves [47]. The coastline of the southeastern bay of the Antofagasta Region is characterized by an extended intertidal rocky platform [48], which harbors an ecosystem with abundant biofilms (i.e., abundant surfaces areas are colonized). In this area, the tides expose living organisms to extreme and often disadvantageous conditions, including highly intensive sunlight, with a monthly average direct solar radiation of ~7000 Wh m^−2^; extremely low rainfall, with an average of 7 mm/year, and high summer air temperatures (20.1 ± 0.1 °C) [49]. In addition, as a result of mining activities, the water has a high load of heavy metals [50]. These extreme conditions make this environment an interesting one for the search of novel microalgal strains tolerant of a wide range of environmental stressors, while at the same time furthering our knowledge about the region’s biodiversity. In this context, the novel strain described here for the first time in Chile, *T. marina* AC16-MESO, which was isolated from a biofilm on the rocky intertidal shore of the Antofagasta Region, is of great interest.

The morphology of the novel strain suggested that it is of the genus *Tetraselmis*, with a characteristic persistent benthic stage, in which colonies adhere to a substrate via a septate pedunculus. These structures have previously been observed in species such as *Prasinocladus marinus* (Cienkowski) [51], which was later re-classified as *Tetraselmis marina* (Cienkowski) [37]. During this sessile phase, cells divide giving rise to daughter cells within the theca. The pedunculus is formed by the successive generation of thecae within the original cell wall of a sessile cell. The cells have the potential for mobility and can swim for a short time before settling and producing a new pedunculated colony [52]. While conducting a microscopy analysis of strain AC16-MESO, all the discussed morphological characteristics were identified. However, the mobile phase was not observed, as the cells remained in their benthic colony state throughout our observations. Molecular analyses of 18S and ITS1-5.8-ITS2 sequences allowed the identification of the strain as *Tetraselmis marina*, thus corroborating the findings from the morphological analysis. Further support for this classification came from the matched ITS-2 secondary structures in *T. marina* AC16-MESO and the other strains of *T. marina*. It is worth noting that our ITS-2 secondary structure analysis also identified the previously unidentified Chilean strain *Tetraselmis* sp. CCM-UDEC 134 isolated in Caldera Bay (27°05′ S; 70°82′ W) by Gonzáles et al. (2015) [25] as *Tetraselmis subcordiforme*. This was based on the secondary structures of both helix II and helix III of the ITS-2 region. Furthermore, the phylogenetic analysis shows the novel strain AC16-MESO as a member of the Chlorodendraceae, and both 18S and ITS1-5.8-ITS2-based trees indicate a close relationship with *T. marina* by grouping the newly isolated strain in a separate clade with other strains of *T. marina*. The results also show a close relationship of *T. marina* with other species of the *Tetraselmis* genus such as *T. cordiformis* and *T. rubens.*

According to the AlgaeBase database [34], *T. marina* has not been reported before from Chile; however, there is a previous report of its presence as *Prasinocladus marinus,* in the same area of origin of the strain described in this study, which researched behavioral responses of *Concholepas concholepas* larvae [53]. In South America, it has only been registered on Argentina’s coast, both as *Prasinocladus marinus* (Cienkowski) Waern [52,54] and under its updated taxonomic label *T. marina* [55]. In North America, it has been reported from New Hampshire as *Prasinocladus marinus* [56] and from California [57,58] and Washington [57] as *Tetraselmis marina*. In Central America, there are reports from Mexico [59,60]. In Europe, it has been reported as *Prasinocladus marinus* from Spain [61], the Baltic Sea [62], and Ukraine [63] and as *Tetraselmis marina* from Germany [64] and Netherlands [65]. There are also reports from Asia (Japan) [57] and Australia and New Zealand [66].

When considering the introduction of a new species to biotechnological applications, it is important to characterize the species growth and productivity, as well as its biochemical properties. Cadoret et al. (2012) compares the high productivity of microalgae from the Atlantic coast, being 10 g m^−2^ day^−1^ versus classic plant crops in Europe, which are 1–2 g m^−2^ day^−1^ [67]. This stark contrast in productivity is of significant importance from the perspective of the biotechnological interest in microalgae. In this respect, the maximum cellular density reached by our novel strain was 8.7 × 10^6^ cells/ml, with an OD540 of 1.3, and the maximum productivity, which was reached in the middle of the exponential phase, was 0.2 g L^−1^day^−1^. This productivity is similar compared to that of other *Tetraselmis species* (0.15–0.2 g L^−1^day^−1^) [68] as well as other chlorophyta like *Chlorella* sp., *Coelastrella* sp., *Scenedesmus* sp., and *Vischeria* sp., collected in Asia, North America, and the Middle East, for which productivity in the range of 0.09–0.17 g L^−1^day^−1^ has been reported [69]. The results of the proximate analysis obtained in culture with f/2 medium (22.3% proteins, 6.6% lipids, and 43.9% carbohydrates) indicate that the composition of the novel strain *T. marina* AC16-MESO is similar to that of the other Tetraselmis strains used in the analysis. However, it should be noted that the composition characteristics are highly variable depending on the culture conditions and media used. For instance, different species within the *Tetraselmis* genus may exhibit diverse composition results. In a study conducted by Khatoon et al. (2018) with the species *Tetraselmis chuii*, protein composition ranged from 26% to 45%, lipids from 16% to 28%, and carbohydrates from 11% to 22% in cultures with Conway medium compared to medium plus wastewater [70]. Another example comes from the study conducted by Batista et al. (2019) on the species *Tetraselmis suecica*, where they obtained a composition of 40.2% proteins, 28.5% lipids, and 10.2% carbohydrates through cultivation in photobioreactors using semi-batch mode with F medium [71]. It should be highlighted that the strain AC16-MESO had the highest carotenoid content, similar to *Nanochloropsis gaditana*, which is used as a source of carotenoids in the aquaculture industry. There are many antecedents of the benefits of the inclusion of carotenoids in the diet in farmed fish, being considered semi-essential nutrients that promote optimal survival and growth [72]. Therefore, these nutritional characteristics provide an extra benefit to the biomass considering the reference *T. suecica* species used in aquaculture. Fatty acid composition analysis showed that the novel strain is rich in different fatty acids, most notably so in palmitic acid, which accounted for 21.81% of total fatty acids, as well as linoleic acid, oleic acid, stearidonic acid, linolenic acid, and EPA, which accounted for 6% of total fatty acids, being similar to those observed in the bibliography for the reference *T. suecica* [73]. Biochemical and nutritional analyses thus showed similarities with the other strains of the genus *Tetraselmis*, which are widely used in aquaculture and better carotenoid contents. Regarding its potential biotechnological use, it should be noted that the presence of a sessile stage is worth highlighting; together with the microalga’s large size, it facilitates the collection of biomass by decantation. Compared to other strains with similar properties, this allows a significant reduction in harvesting costs, since it is estimated that collection and dewatering equipment can cost 90% of the total cost to produce algal biomass from open ponds [74].

*T. marina* AC16-MESO tolerated a wide range of salinities, allowing it to grow at varying water quality. Its optimum range was between 5 and 15‰, at salinity levels that were much lower than optimum levels of 20 to 60‰ for biomass production for *T. suecica* [75], as well as optimum levels found by Bartley et al. (2013) for *Nannochloropsis salina*, which are 22 to 50‰. While the concept of cellular stress is not new, Bartley et al. (2013) [76] highlighted that the modification of salinity levels in order to stress microalgal cells is a key to increasing lipid production in the phases of cellular growth. Specifically, lipid production is mediated through three metabolic pathways which assimilate carbon: (i) incorporation of photosynthetic CO_2_ into fatty acids inside chloroplast; (ii) starch degradation, and (iii) polar lipids degradation, however, in order to clarify which or how this could be happening in the present microalgae, transcriptomics analyses are required [77,78,79]. Lipid accumulation due to high salinity has also been achieved by other microalgae such as *Chlamydomonas* sp. [77], *Chlorella vulgaris* spp. [80,81], *Scenedesmus* spp., and *Chlorella* sp. strains [82], among others. Here, a considerable increase in the level of lipids was observed under saline stress, at both hypo and hypersaline conditions.

Along with all these aforementioned characteristics, the capacity to increase lipid content also harbors potential for the production of biofuels, as observed in other *Tetraselmis* species [18,19]. Additionally, the high carbohydrate content in the microalgal strain provides a valuable resource for various biotechnological applications, such as bioethanol, biobutanol, biomethane, bioplastics production, and other valuable compounds like prebiotics [83,84]. On the other hand, the potential use of this strain as a feedstock for aquaculture has been discussed, attributed to its similar content compared to other *Tetraselmis* species [24] and high carotenoid content [72]. Regarding other potential biotechnological applications, it is important to highlight the results obtained by the research group concerning its tolerance and potential use in heavy metal contamination remediation. The strain has shown high removal efficiency of Cu^2+^, Fe^3+^, and Mn^2+^ as well as its potential as a bioabsorbent to remove Zn^2+^ from industrial effluents [17,85].

## 4. Materials and Methods

### 4.1. Isolation and Culture of the Microalga

The study site from which the samples were collected is located on the coast of the Antofagasta Region (Chile; approximate coordinates: 23°36′57.2″ S, 70°23′33.8″ W). Samples were taken from the biofilm covering intertidal rocks by swabbing with a sterile cotton bud and subsequently cultured in liquid Guillard’s (f/2) medium [86] based on 0.2 micron-filtered and autoclaved seawater. From there, they were seeded onto agar plates containing the same medium. Clones were obtained from diverse species of microalgae, but based on morphological and growth-related characteristics, the strain AC16-MESO was selected for a more detailed characterization. Monoalgal colonies were transferred to new solid medium plates and successively re-seeded until obtaining a monospecific culture of the strain of interest. This monoalgal culture was then maintained in Guillard’s (f/2) medium at 20 °C, under photon irradiance of 80 µmol m^−2^ s^−1^ and with a 12 h:12 h light–dark photoperiod, in the culture collection of the Mesoscosmos Marino Laboratory at the University of Antofagasta.

### 4.2. Optical and Electronic Microscopy

- Bright-field and epifluorescence microscopy

For both bright-field and epifluorescence microscopy, cells were observed alive, and, for lipid detection, after Nile Red staining, with an Olympus BX 51 microscope fitted with an Olympus DP70 digital camera and using the Image-Pro software (v11).

- Scanning electron microscopy

For visualization under the scanning electron microscope (SEM), the microalgae were collected on 450 µm nylon membranes (Fabric Nylon Sefar Switzerland Num 42GR GG AB). Membrane pieces measuring 1 cm × 1 cm were transferred into Eppendorf tubes containing 2 mL of culture medium. Fixation was achieved by adding 160 µL of 25% glutaraldehyde for a final concentration of 2% glutaraldehyde and letting the mix stand at 5 °C for a week. The fixative was then washed off in the following sequence (each washing step lasted 15 min): three washes with culture medium, two washes with a 3:1 mix of medium and distilled water, two washes with a 1:1 mix of medium and distilled water, two washes with a 1:3 mix of medium and distilled water, and five washes with distilled water. Following this, the samples were dehydrated in a series of dilutions of ethanol (20 min each in 10%, 25%, 50%, 75%, and 100% ethanol at room temperature), critical point-dried (BalTec, Reading, UK) and mounted on an aluminum SEM stub. Finally, the samples were coated with gold/palladium and observed under a Hitachi 3000 scanning electron microscope.

### 4.3. PCR Amplification, Sequencing, and Identification of the Microalgal Strain

- DNA extraction and amplification

Genomic DNA was extracted in triplicate using the PowerSoil^®^ MoBio extraction kit (MoBio Laboratories). The 18S ribosomal DNA fragment was amplified using the primer pairs: NS1 forward (5′-GTAGTCATATGCTTGTCTC-3′) and NS8 reverse (5′-TCCGCAGGTTCACCTACGGA-3′) [87]. The ITS1-5.8-ITS2 fragment was amplified using the primers ITS forward1 (5′-ACCTAGAGGAAGGAGAAGTCGTAA-3′) and ITS Reverse1 (5′-TTCCTCCGCTTATTGATATGC-3′) [88]. PCR amplifications used 100 ng of extracted DNA in a final reaction volume of 30 μL, with 5 μL 10× Green Buffer, 0.5 µL 10 mM dNTPs, 2 μL 25 mM MgCl_2_, 1 μL of each primer at 10 mM, and 0.25 µL GoTaq DNA polymerase (0.23 U/µL, PROMEGA Corporation, Madison, WI, USA). The PCR program was as follows: initial denaturation at 95 °C for 5 min, followed by 35 cycles of denaturation at 94 °C for 30 s, annealing at 55 °C for 1 min and elongation at 72 °C for 1 min, and a final elongation step at 72 °C for 5 min. PCR products were purified using the UltraCleanTM15 DNA kit (Mobio Laboratories, Carlsbad, CA, USA) following the manufacturer’s instructions and stored at 4 °C.

- Sequencing and identification

The purified PCR products were sequenced at Macrogen Inc. (Seoul, Republic of Korea) on a 3730xl DNA Analyzer automatic sequencer. Sequences were analyzed, cleaned, and aligned using the program Chromas Pro. The sequences obtained were analyzed with the GenBank using BLAST (www.ncbi.nlm.nih.gov/blast/Blast.cgi, accessed on 15 June 2023) in order to identify the species of the new strains. Sequences obtained for 18S ribosomal DNA and the ITS1-5.8-ITS2 region were deposited in GenBank under the accession numbers MN304931 and KX752433, respectively.

### 4.4. Phylogenetic Analysis

Two independent phylogenetic trees were generated for the 18S gene and the ITS1-5.8-ITS2 region. The first phylogenetic tree was constructed by aligning the sequence of the 18S ribosomal gene (1617 bp) and the second tree by aligning the sequences of the ITS1-5.8-ITS2 region (612 bp) using the MUSCLE algorithm [89], including some other homologues species of *Tetraselmis* genus. The structure of the tree was assembled using the maximum likelihood algorithm with the Tamura-Nei nucleotide substitution model (previously checked with jModelTest 2.1.10 v20160303 software) and a bootstrap replicate value of 100. All the previously mentioned analyses were conducted using Geneious Prime v2009.0.4 software with default settings [90,91,92]. The target organism is highlighted with bold font. The bootstrap proportion is shown next to every node and the lower bar indicates relative genetic distance. The NCBI ID is being shown after every name of each organism. The organism *Chlamydomonas applanata* (accession number FR865570) and *Chlamydomonas reinhardtii* (accession number JX839534) were used as the outgroup for the analysis of the 18S and ITS sequence, respectively.

### 4.5. ITS-2 Secondary Structure

The secondary structures of ITS-2 rDNA helices II and III were modeled for the newly isolated AC16-MESO strain, as well as for the other strains of interest, using the program mfold [93]. The following eight strains were used in the comparative analysis of secondary structures: *Tetraselmis marina* IOAC331S (accession number KC800942.1), *Tetraselmis marina* CCMP898 (accession number HE610131), Tetraselmis subcondiformis (accession number KC137971), *Tetraselmis striata* SAG 41.85 (accession number HE610129), *Tetraselmis suecica* WDCM NCC62 (accession number AY574382), and the strains *Teatraselmis* sp. CCM-UDEC 109 (accession number KF250349), *Tetraselmis* sp. CCM-UDEC 114 (accession number KF250348), and *Tetraselmis* sp. CCM-UDEC 134 (accession number KF250350), which were isolated from the Chilean coast by González et al. [25] (2015). RNA processing sites and compensatory base changes (CBCs) were identified manually.

### 4.6. Growth Characteristics in f/2 Medium

The growth characteristics of the microalga were analyzed by establishing its growth curve in f/2 medium [77]. Cultures were set up in 2l bioreactors under constant bubbling of air through the lower part of the reactor, at a controlled temperature of 20 °C and in a simulated daily light cycle. The microalga was added at a concentration of 250,000 cells/mL, and its growth was monitored over 20 days in triplicate and run in separate bioreactors. Every other day, dry weight was estimated, a cell count was performed in a Neubauer chamber, and, as a measure of cellular density, the optical density at 540 nm was measured in a spectrophotometer. Absorbance at 540 nm was correlated with the number of cells per volume (mL). The linear relationship between microalgal density and OD540 is given by Equation (1):

y = 0.1276x + 0.0791 (R^2^ = 0.94), (1)

where x represents cellular density in units of millions of cells and y represents OD540.

The biomass for the dry weight estimation was obtained by filtering with a 0.45 µm GF-C fiberglass filter (WHATMAN) and drying in an incubator over 48 h prior to estimating the biomass in g/L.

Biomass productivity was also measured and expressed in g L^−1^ day^−1^, as indicated by Equation (2):

Pb = (DWt2 − DWt1)/t2 − t1,(2)

where DWt2 and DWt1 are the dry weight calculated at times t2 and t1, respectively.

### 4.7. Proximate Analysis, Analysis of Pigments, and Fatty Acid Composition

For the analysis and comparison of the biochemical composition of the newly identified strain, reference microalgae of genera frequently used in aquaculture were used, such as: *Nannochloropsis* and *Tetraselmis*. Specifically, these strains were used: *Nannochloropsis gaditana* (CCMP 527) and *Tetraselmis suecica* (strain belonging to the collection stock of the Applied Microbiology Unit of the University of Antofagasta). These were cultivated in the same facilities and culture medium. The biochemical composition of the biomass of each strain was determined at the end of the growth curve experiment, after harvesting, centrifuging, and lyophilizing microalgal cells.

The protein content was quantified using the modified Lowry method proposed by Herbert et al. (1971) [94]. Total lipids were determined according to Kochert (1978) [95]. The ash content was measured by incineration of a 100 mg sample in an oven at 450 °C for 48 h. The carbohydrate content was estimated by subtracting the sum of the other fraction percentages (ash, lipids, and proteins) from 100. Chlorophyll content was determined by the method of Mackinney [96], after disruption of cells by sonication for 5 min and extraction with methanol at 40 °C for 1 h. Total carotenoid content was determined spectrophotometrically by the method of Davies [97], after disruption of cells by sonication for 5 min and extraction with acetone for 3h. The content and composition of fatty acids was determined by transesterification and gas chromatography (Agilent Technologies 6890 N Series Gas Chromatograph, Santa Clara, CA, USA), as described by Rodríguez-Ruiz et al. [98].

### 4.8. Salinity Tolerance Assays

In order to test the microalga’s tolerance of salinity, cell counts were monitored using a Neubauer chamber at 72, 96, 120, and 168 h of culture at salt concentrations of 0.6, 1.25, 2.5, 5, 15, 30, 45, 60, 90, 120, and 35 (control) (‰). The bioassay was performed in multiwell plates starting from the inoculation of 3 mL of f/2 medium [77] or distilled water (autoclaved at 121 °C) with 3 × 10^5^ cells/mL. All treatments were applied in triplicate, and the cultures were incubated in a controlled environment at 20 ± 1 °C in a 12 h light: 12 h dark cycle.

### 4.9. Photosynthetic activity

Photosynthetic activity was determined through the in vivo chlorophyll a fluorecence of photosystem II (PSII) using a chlorophyll fluorometer (JUNIOR-PAM, WALZ, Germany). The optimal quantum yield of photosystem II (Fv/Fm), which serves as an indicator of photosystem II integrity in the microalga, was monitored in triplicate at 72, 96, 120, and 168 h during the salinity tolerance assay after 10 min of adaptation of the microalgae to darkness and was calculated according to Schreiber [99]:

Fv/Fm = Fm − F_0_/Fm, (3)

where Fv is the variable fluorescence, Fm is the maximum fluorescence, and F_0_ is the minimum fluorescence.

### 4.10. Determination of the Lipid Content in Stressful Salinity Situations

The effects on the lipid content of stress under different salinity conditions were evaluated in the same conditions indicated in the previous section at 72, 96, 120, and 168 h of culture at salt concentrations of 0.6, 1.25, 2.5, 5, 15, 30, 45, 60, 90, 120, and 35 (control) (‰). The method of Chen et al. [100], which involves staining with Nile Red (9-diethylamino-5-benzo[α]phenoxazinone) for the detection of polar and neutral lipids in fluorometry assays, was employed. The assay was performed in 96-well plates containing 300 µL 25% DMSO per well standardized to 100,000 cells/mL using a Neubauer chamber and stained with Nile Red at a final concentration of 1 µg/mL. The plate was incubated at 40 °C for 10 min. The fluorimetry assay was carried out using a GloMax^®^-Multi detection system (PROMEGA Corporation, Madison, WI, USA) at an excitation wavelength of 490 nm and an emission wavelength of 510–570 nm. A 25% solution of DMSO with 1 µg/ml Nile Red was used as a blank.

### 4.11. Statistical Analysis

All parameter measurements and assays were carried out in triplicate, and means ± standard deviations were calculated. The resulting values were compared in an analysis of variance (ANOVA) with a 95% Tukey test. Differences were considered significant at a threshold of *p* < 0.05. Statistical analyses were carried out using the program GraphPad Prism version 5.01.

## 5. Conclusions

The new microalgal strain, AC16-MESO, isolated from the coast of Antofagasta, Chile, and identified in this study as *Tetraselmis marina*, represents the first report of this species in Chile that includes morphological, molecular, and biochemical characterization. Its biochemical and nutritional profile, size combined with the presence of a sessile state, and the consequent ease of collection by decantation, added to a high tolerance to a wide range of salinities and heavy metals, make this strain a promising candidate for use in biotechnology, with qualities for use in bioremediation as well as a potential source of compounds of interest and as feed for aquaculture.

## Figures and Tables

**Table 1:** Fatty acid (FA) composition of new microalgal species *T. marina* AC16-MESO. Values represent the mean ± SD of three independent measurements (*n* = 3).

*Tetraselmis* sp. AC16-MESO														
	C16	16:1n7	16:2n4	16:3n4	18:1n9	18:1n7	18:2n6	18:3n3	18:4n3	20:1n9	20:4n6	20:5n3	Others	FA, % d.wt
**%FA with respect to the total**	**22.5** ± 0.89	**1.2** ± 0.03	**4.7** ± 0.71	**0.7** ± 0.05	**8.8** ± 0.23	**1.8** ± 0.73	**11.2** ± 2.00	**9.1** ± 1.37	**7.9** ± 0.86	**2.7** ± 1.47	**2.1** ± 0.13	**6.1** ± 0.84	**25.1** ± 4.26	**4.2** ± 0.28

**Table 2:** Fv/Fm values for *T. marina* AC16-MESO in response to stress treatments at different salt concentrations. The control is microalgae *T. marina* AC16-MESO in sea water (35‰). Values represent the mean ± SD of three independent measurements (*n* = 3).

	Salinity (°%)										
Time (hrs.)	Control	0.6	1.25	2.5	5	15	30	45	60	90	120
72	0.71 ± 0.01	0.66 ± 0.01	0.65 ± 0.01	0.69 ± 0.01	0.70 ± 0.00	0.70 ± 0.01	0.63 ± 0.03	0.59 ± 0.02	0.58 ± 0.01	0.54 ± 0.02	0.16 ± 0.05
96	0.67 ± 0.01	0.48 ± 0.08	0.51 ± 0.02	0.66 ± 0.00	0.62 ± 0.06	0.66 ± 0.03	0.55 ± 0.05	0.52 ± 0.03	0.49 ± 0.03	0.51 ± 0.01	0.08 ± 0.03
120	0.59 ± 0.03	0.40 ± 0.16	0.49 ± 0.01	0.47 ± 0.01	0.55 ± 0.06	0.57 ± 0.03	0.54 ± 0.04	0.52 ± 0.01	0.46 ± 0.02	0.50 ± 0.01	0.05 ± 0.02
168	0.49 ± 0.04	0.31 ± 0.07	0.36 ± 0.03	0.48 ± 0.01	0.45 ± 0.02	0.52 ± 0.05	0.39 ± 0.05	0.38 ± 0.08	0.30 ± 0.02	0.30 ± 0.01	0.04 ± 0.01

## Data Availability

Not applicable.
